# Case Reports on Metaplastic Squamous Cell Carcinoma of the Breast and Treatment Dilemma

**DOI:** 10.1155/2019/4307281

**Published:** 2019-09-18

**Authors:** Anita Pandey, Kishor Joshi, Harry Moussouris, Gardith Joseph

**Affiliations:** ^1^Department of Hematology and Oncology, Brookdale University Hospital Medical Center, USA; ^2^Department of Internal Medicine, Brookdale University Hospital Medical Center, USA; ^3^Department of Pathology, Brookdale University Hospital Medical Center, USA

## Abstract

Metaplastic squamous cell carcinoma of the breast is a very rare form of breast cancer that consists of both glandular and nonglandular components mixed with epithelial and mesenchymal tissues. Worldwide, the incidence of this tumor is between 0.1 and 2%. Because of the rarity of this tumor and heterogeneous behavior of the tumor cells, it is difficult to establish the standard therapeutic approach. We report 2 cases of metaplastic squamous cell carcinoma of the breast in young patients with different responses to treatment strategies. The first case is a premenopausal female with metaplastic squamous cell carcinoma treated with surgery, chemotherapy, and radiotherapy, and the second case is perimenopausal metaplastic squamous cell carcinoma with sarcomatoid subtype and osteoid matrix production which progressed on chemotherapy and was treated with surgery and radiation.

## 1. Introduction

Squamous cell carcinomas (SCC) are common in the skin and respiratory and upper GI tracts lined by squamous cells. Metaplastic breast carcinoma is a rare malignancy [1, 2]. The Surveillance, Epidemiology, and End Results (SEER) database of the National Cancer Institute (NCI), from 2011 to 2015, shows a total of 168 cases of epidermoid carcinoma which accounted for 0.1% of total invasive breast carcinoma [3]. Epidermoid carcinoma includes squamous, basal, and transitional cell carcinomas. It was not recognized as a distinct entity till 2000. WHO 2012 classified metaplastic breast carcinoma as invasive breast cancers with squamous or mesenchymal components with elements of spindle, chondroid, osseous, and rhabdomyoid cells mixed with the usual cell type [4]. Depending on the cellular behavior, it can be either low-grade tumors (low-grade adenosquamous carcinoma or low-grade spindle cell carcinoma) or high-grade tumors (high-grade squamous cell or high-grade spindle cell).

Broadly, it is categorized under 3 categories: first, metaplastic carcinoma of no specific type that includes low-grade adenosquamous carcinoma, squamous cell carcinoma, spindle cell carcinoma, and fibromatous-like metaplastic carcinoma; second, metaplastic carcinoma with mesenchymal differentiation that includes chondroid differentiation, osseous differentiation, and other types; and third, the mixed type. When the squamous cell component (SCC) predominates by more than 90%, they are pure squamous cell carcinoma and tend to be more aggressive and treatment refractory [5]. For confirmation of a diagnosis of primary SCC of the breast, the following three criteria must be fulfilled: absence of an associated primary SCC in a second site, the absence of skin involvement, and a clear predominance (>90%) of areas with SCC at histologic examination.

There are different hypotheses to explain the histogenesis of squamous cell carcinoma of the breast. It may arise de novo from epithelium lining of the breast or present as a small foci in preexisting adenocarcinoma or deep-seated epidermal cyst [6, 7].

In either case, they express heterogeneous somatic mutations [8, 9]. These mutations are different from that of triple negative breast cancer. In one of the study aberrations on PIK3CA/PIK3R1 and Ras-Map kinase pathway in 61% and 25%, respectively, in addition to increased frequency of TP53 (64%) and TERT promoter (25%), mutations in comparison to triple negative carcinomas have been reported. In the other study, metaplastic breast cancer was found to harbor complex mutations leading to activation of the PI3K/AKT/mTOR pathway (57% vs. 22%) and canonical Wnt pathway (51% vs. 28%) as opposed to triple negative breast cancer. Other additional mutations like ARID1A (11%), FAT1 (11%), PTEN (11%), PIK3CA (29%), and PIK3R1 (11%) are expressed in comparison to TN breast cancer. Interestingly, it was found that PIK3CA mutations were absent in MBCs with chondroid metaplasia [10]. Though they have very poor prognosis, the determinant of the prognosis is not clear.

## 2. Case No. 1

**Figure 1:**
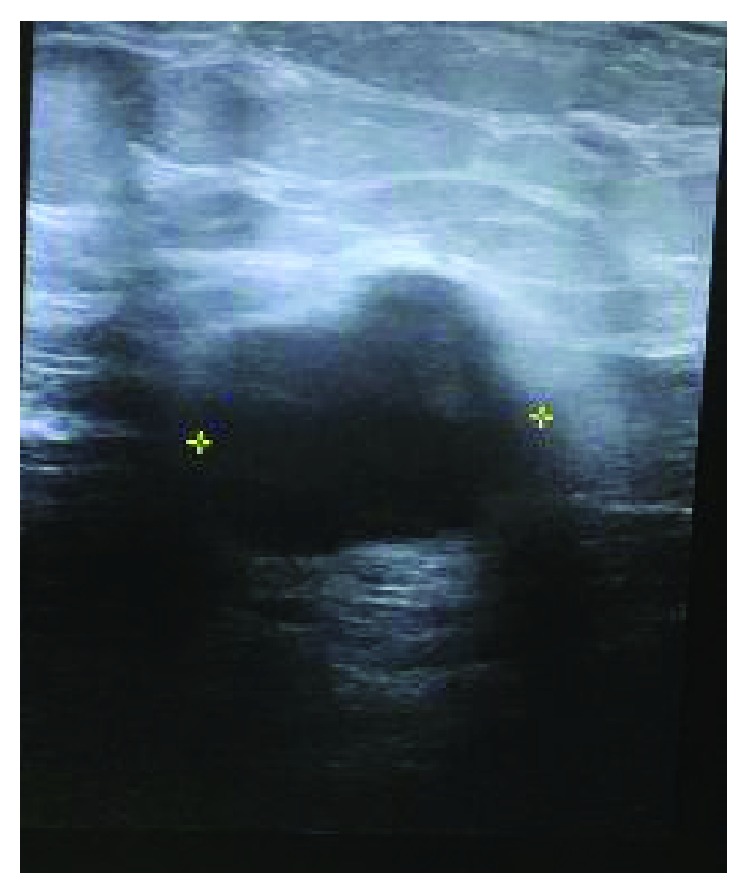
Sonogram of the left breast showing hypoechoic lesion.

**Figure 2:**
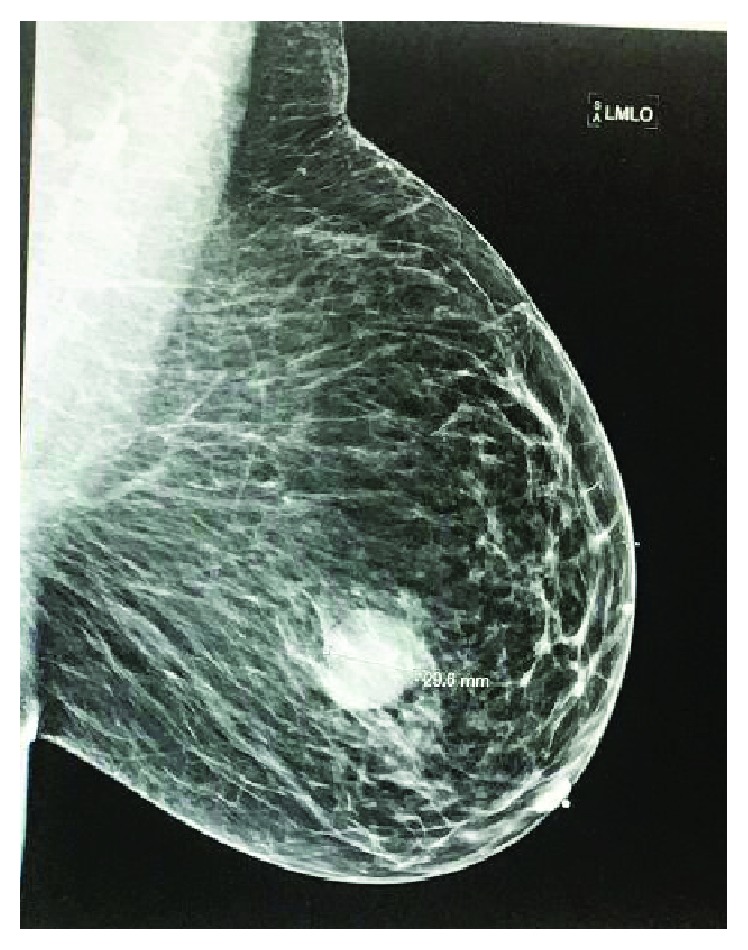
Mammogram of the left breast.

**Figure 3:**
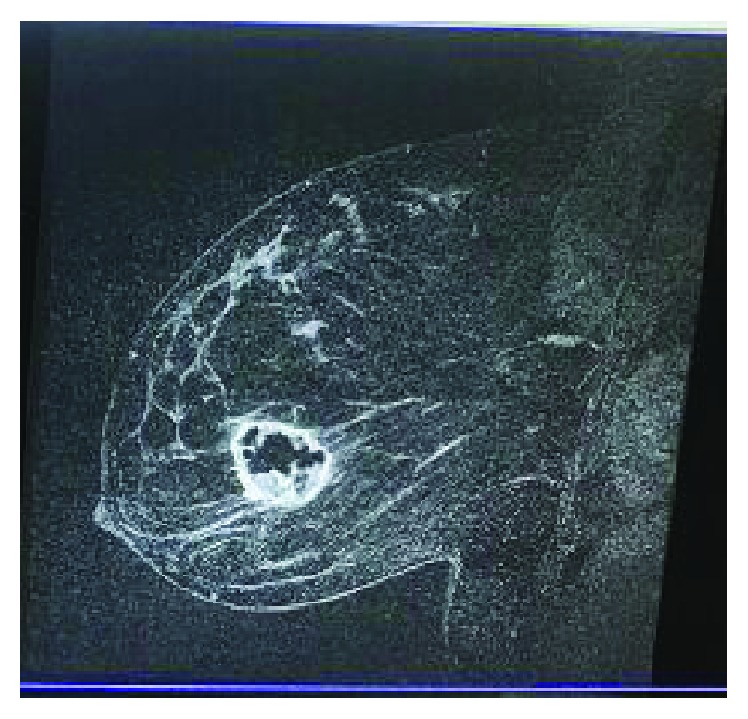
MRI left breast.

**Figure 4:**
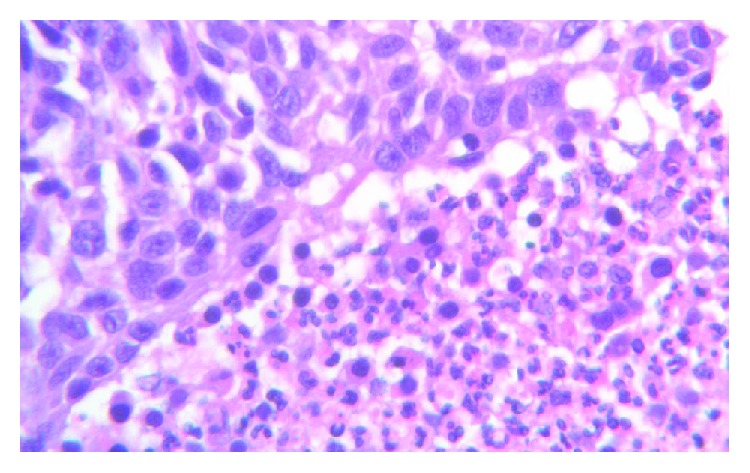
High-grade features with cell atypia.

**Figure 5:**
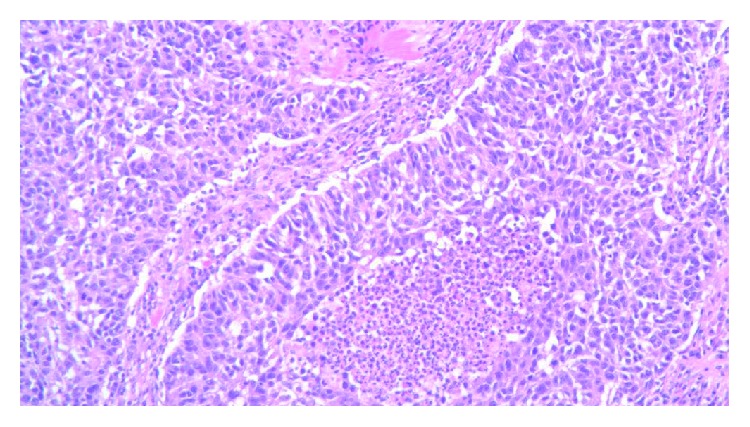
Cell atypia with necrosis.

A 39-year-old African American female presented to her primary care physician with a palpable lump in her left breast. Her past medical history includes hypertension, diabetes mellitus, hyperlipidemia, and seizure. She smoked half a pack of cigarette for the past 16 years. The family history was noncontributory. Ultrasound (Figure 1) and mammogram (Figure 2) of the left breast reported a 2.5 cm lobulated hypoechoic lesion with well-demarcated borders and posterior acoustic enhancement suggestive of a complicated cyst. The bilateral breast MRI (Figure 3) that was done 3 weeks later revealed a left retroareolar 3.8 × 3.7 × 3.5 cm mass complex, heterogeneous with rim enhancement. No other mass or lymphadenopathy was seen. An ultrasound-guided biopsy revealed triple negative, moderately differentiated invasive left breast ductal carcinoma. The right breast was unremarkable. She underwent modified radical mastectomy and lymph node dissection with tissue expander placement 6 weeks after her first ultrasound. The final pathology revealed a 5.5 cm primary invasive metaplastic ductal carcinoma (Figure 4). The histologic grade was 3 with squamous differentiation and was triple negative (Figure 5). Immunohistochemistry was positive for E-cadherin and focally positive for GATA which indicated ductal origin. p63 and cytokeratin 5/6 were positive in favor of metaplastic squamous cell differentiation. Proliferative index Ki-67 was >40%. All of the six nodes were negative for a tumor. The rapid growth of the tumor from the time of the diagnosis to the time of surgery explains the aggressive nature of this group of the invasive disease. The final stage was IIB (T3N0MX) metaplastic breast cancer. The patient received adjuvant chemotherapy with dose-dense AC-T (Adriamycin and cyclophosphamide followed by paclitaxel). After completion of chemotherapy, the patient underwent whole breast radiotherapy. Eight months post treatment, the patient is in remission and has no signs of recurrence.

## 3. Case No. 2

**Figure 6:**
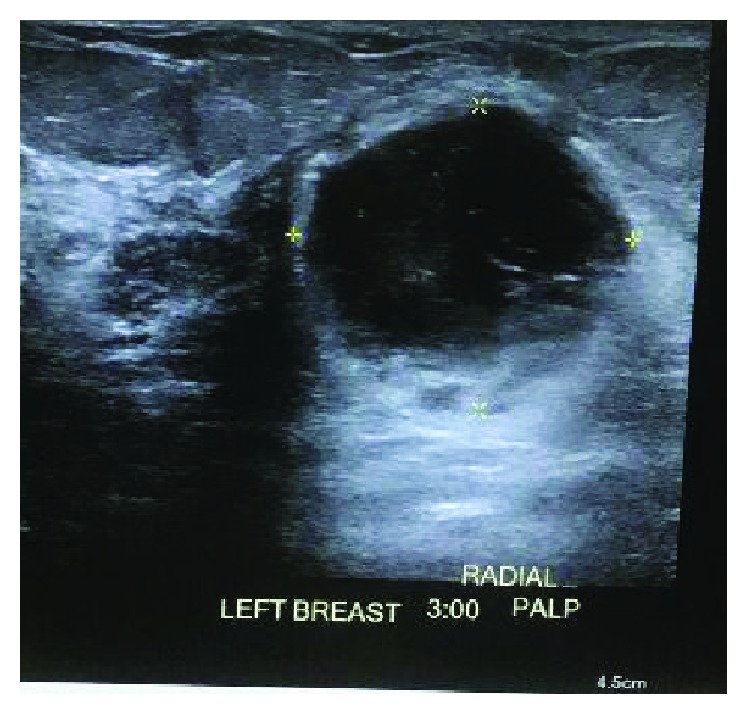
Left breast sonogram showing a cystic mass.

**Figure 7:**
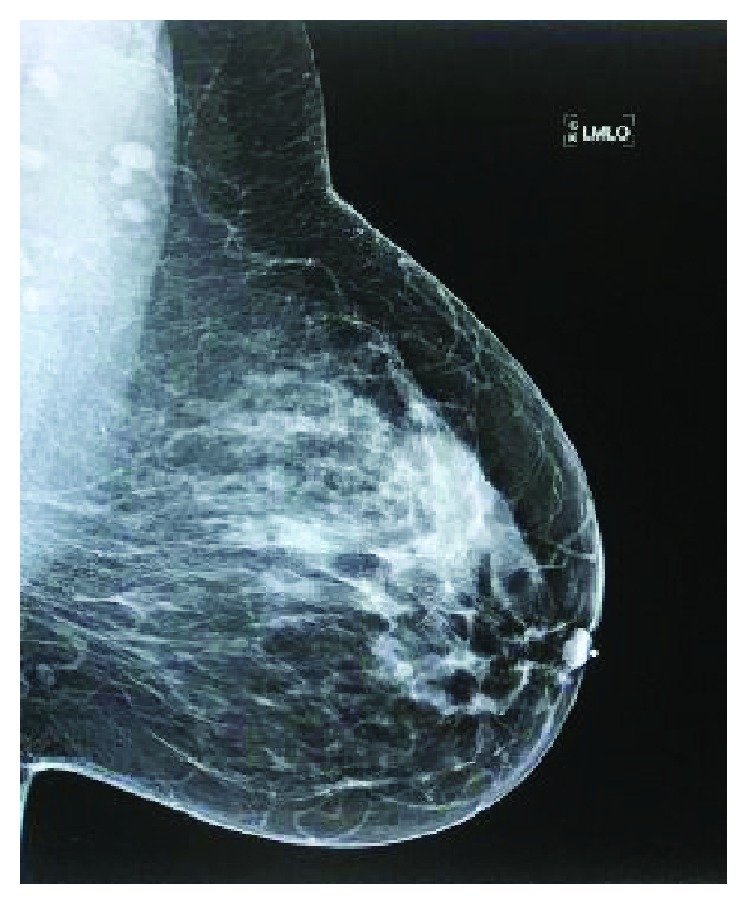
Left breast mammogram.

A 53-year-old female with a history of abdominal extra-adrenal paraganglioma, status postsurgical removal, endometriosis, hypothyroidism, HTN, and thyroid nodule noticed a left breast lump for a month during her breast self-exam. On the physical exam at the oncology office, her breast was symmetrical. There were no skin or nipple changes. A mass of 2 × 2 cm and 5 cm from the nipple at 5:00 PM was appreciated with no evidence of axillary lymphadenopathy. She promptly underwent a sonogram (Figures 6 and 7) and mammogram that revealed a heterogeneous breast, and corresponding to the palpable abnormality was a large complex cystic mass measuring 2.7 × 2.4 × 2.6 cm with a thick wall with enhanced through transmission and some internal vascularity. She underwent lumpectomy. The gross specimen was described as a 5.5 × 5 × 5 cm mass with a centrally located cystic hemorrhagic tumor which measured 3.3 cm in the greatest dimension and negative margin. It was reported as high-grade metaplastic carcinoma (3.3 cm), with sarcomatoid subtype and osteoid matrix production, and high-grade DCIS. Immunostaining for estrogen and progesterone receptors and HER2 was negative. The Ki-67 proliferation index was markedly elevated to 90%. The area of squamous cell carcinoma was positive for p63 and p40. The sarcomatoid area was negative for cytokeratin (CK AE1/3).

**Figure 8:**
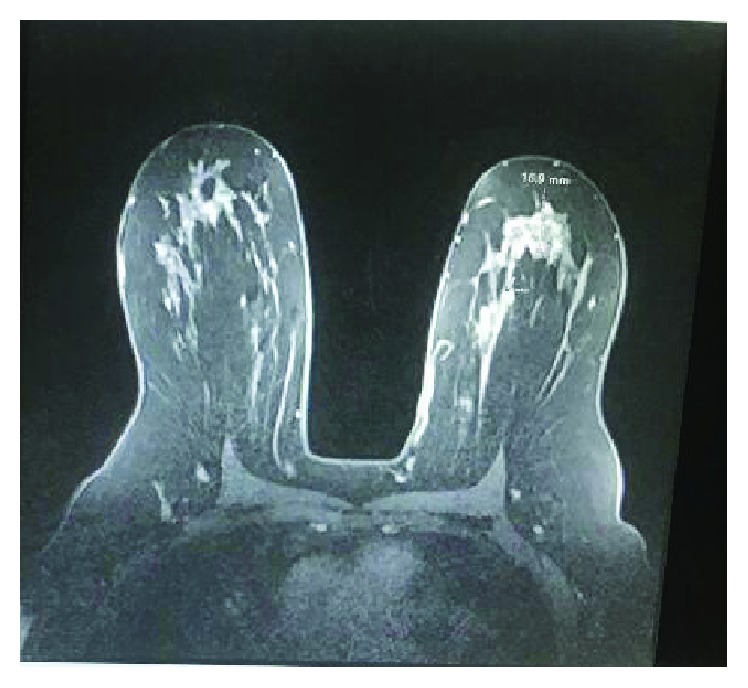
MRI left breast.

On her follow-up clinic visit at 4 weeks post lumpectomy, the mass was felt on the surgical site on the physical exam. MRI bilateral breasts showed an enhancing mass of maximal dimension of 1.7 cm and subtle asymmetric left axillary tail lymph node 1.4 × 0.9 cm (Figure 8). Staging CT chest/abdomen/pelvis was negative except for the left breast mass. The patient underwent mastectomy and axillary lymph node dissection.

**Figure 9:**
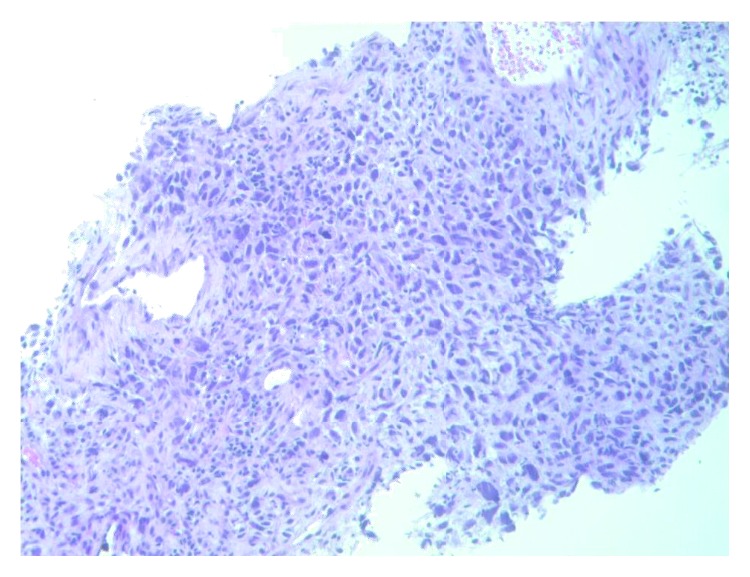
Cells with sarcomatoid differentiation.

**Figure 10:**
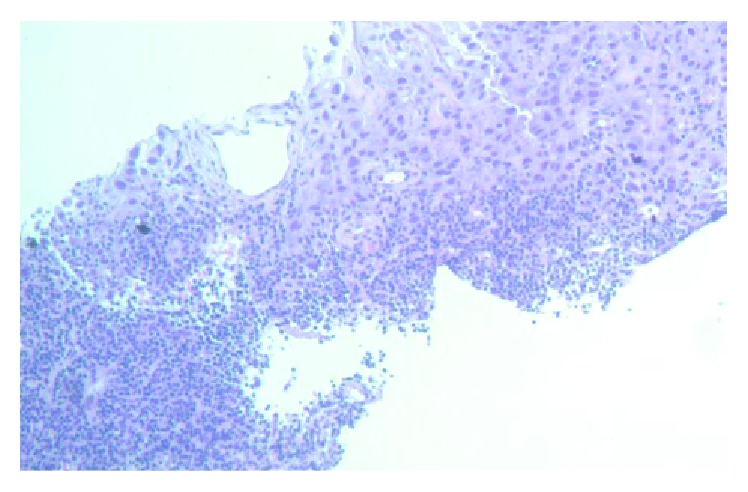
Lymph node biopsy—lymphocyte aggregates and adjacent tumor cells.

Post mastectomy, the gross specimen was reported as a firm, cystic, hemorrhagic tumor with 4.8 × 4.5 and 4 cm in dimension located 2.5 cm from the closest inked margin. Pathology description was invasive breast cancer with osteoid formation (metaplastic) features, grade 3 with no lymph vascular invasion and negative margin, and DCIS 0/15 lymph node was negative for malignancy. Hormonal receptor and HER2 status were negative. The patient was scheduled to start on dose-dense 4 cycles of AC (Adriamycin and cyclophosphamide) followed by paclitaxel and carboplatin. After 2 cycles of AC, she was admitted with a chest pain. CT chest showed a new mass 3.9 cm complex nodule within the left axilla and 1.8 cm circumscribed nodule within the lateral aspect of the left breast. The biopsy of the lymph node was suggestive of high-grade sarcoma (Figures 9 and 10) with a positive stain for AE1/AE3, CK7 (rare), p63 (rare), CDX2 (focally weak), GATA-3 (rare), and PAX-8 (weak) and negative for CK20, TTF1, Napsin A, S100, GCDFP-15, ER, CA125, mammaglobin, and CEA monoclonal. An additional stain was negative for CD117 and focally positive for DOG1.

The patient underwent further resection of the chest wall mass and axillary lymph node. Pathology disclosed a gross specimen of 1.8 cm chest wall lesion and 3.0 × 3 × 2.5 cm axillary mass with four additional nodules up to 0.5-0.7 cm with histological similarity to the first tissue biopsy and Ki of >90. Next generation sequencing was negative for any mutations. Post resection, she received radiation without any signs of recurrence.

## 4. Discussion

It is a well-established fact that metaplastic breast cancer is one of the rare entities.

The pathogenesis of metaplastic squamous cell carcinoma of the breast has evolved in the recent few years as our understanding of genetic and nongenetic heterogeneity affecting phenotypical behavior of this type of cancer has broadened. Some authors suggest that it originates from squamous metaplasia [11–14] which is found in the epithelium of the cyst, fibroadenomas, phyllodes tumors, or papillomas or chronic abscess [15] whereas others believe that it arises from myoepithelial cells. Cases were reported of squamous cell carcinoma on a patient with previous history of chemotherapy and radiation chemotherapy for breast cancer [16] and with breast implants [17].

In general, DNA array and immunohistochemistry are performed in addition to histopathology to subdivide breast cancer into 3 broad categories: basal, luminal, and HER overexpression. Though metaplastic squamous cell carcinoma is mostly hormone negative [18], specific immunostaining is unknown. In the past, there have been some attempts to understand the molecular biology of this group of cancer which would help clarify the treatment approach [19]. Pure and metaplastic SCC resemble phenotypically to basal origin: they never expressed ER (estrogen receptor) or PR (progesterone receptor); are HER2-negative in 93% of cases; exhibited positivity for CK 5/6 (cytokeratin) and EGFR in 75% and 85%, respectively, and p63 in 70% of cases; and display a high proliferative index. However, expression profile of SCC of the breast was markedly different from that of IDC (invasive ductal carcinoma). Additionally, it was found that HPV infection is not associated with SCC of the breast.

Metaplastic breast cancer harbors different complex mutations. Recently, aberrations in the PIK3/AKT/mTOR pathway are found more frequently in mesenchymal subtype of metaplastic breast cancers. Clinical trials targeting this pathway in combination with chemotherapy have demonstrated very good response [20–22].

Up to 30% of the tumor is found to have lymph node positive even though lymphatic spread is rare. The SCC of the breast present as a larger primary tumor with higher histological grade and lower incidence of axillary node involvement. Radiologically, no typical mammographic appearances are found and they lack microcalcifications in most cases [23, 24]. Cystic lesions are characteristic presentations of SCC in more than 50% of cases. The definite role of PET CT on squamous breast cancer has not been defined yet [25, 26].

Due to rarity of this tumor, the most appropriate therapeutic regimen for SCC of the breast is not defined [27]. Till date, there is no specific guideline for the treatment [28]. Though chemotherapy for metaplastic breast cancer largely aligns with invasive ductal cancer, the 3-year disease-free survival rate is poor in comparison to IDC [29]. Due to heterogeneity of the tumor, the historical therapy has failed to achieve a prolonged response [30]. Resistance to chemotherapy is likely due to complex genetic and nongenetic makeup. So far, the response to chemotherapy has been different from case to case, and some respond with neoadjuvant chemotherapy [31, 32].

Overexpression of EGFR was found in a metaplastic breast [33] potentiating the role of protein kinase inhibitors against EGFR.

As mentioned earlier, PIK3 inhibitors and mTOR inhibitors in mesenchymal metaplastic breast cancer have shown promising results in early-phase trials.

## 5. Conclusion

Metaplastic squamous cell breast cancer is a rare malignancy. It appears large and cystic with the absence of microcalcifications on imaging. It usually has a higher histological grade and negative hormonal and HER2 status with lower incidence of axillary node involvement. Though surgery has been a standard treatment for localized disease, we have not been able to achieve the same consensus in regard to chemotherapy. In our reported cases, surgery was the mainstay of treatment while the response to chemotherapy was seen in the 1st case as opposed to the second case which progressed while on chemotherapy. As we wait for clinical trials for MBC, treatment should be individualized in context to histology subtypes for better survival.
